# Long distance homing in the cane toad (*Rhinella marina*) in its native range

**DOI:** 10.1242/jeb.243048

**Published:** 2022-01-28

**Authors:** Daniel A. Shaykevich, Andrius Pašukonis, Lauren A. O'Connell

**Affiliations:** 1 Stanford University, Department of Biology, 371 Jane Stanford Way, Stanford, CA 94305, USA; 2 CEFE, Université de Montpellier, CNRS, EPHE, IRD, 34293 Montpellier, France

**Keywords:** Amphibians, Animal movement, Space use, Tracking, Translocation, Navigation

## Abstract

Many animals exhibit complex navigation over different scales and environments. Navigation studies in amphibians have largely focused on species with life histories that require accurate spatial movements, such as territorial poison frogs and migratory pond-breeding amphibians that show fidelity to mating sites. However, other amphibian species have remained relatively understudied, leaving open the possibility that well-developed navigational abilities are widespread. Here, we measured short-term space use in non-territorial, non-migratory cane toads (*Rhinella marina*) in their native range in French Guiana. After establishing site fidelity, we tested their ability to return home following translocations of 500 and 1000 m. Toads were able to travel in straight trajectories back to home areas, suggesting navigational abilities similar to those observed in amphibians with more complex spatial behavior. These observations break with the current paradigm of amphibian navigation and suggest that navigational abilities may be widely shared among amphibians.

## Abstract (precis)

**Summary:** Translocation-homing experiments reveal that non-territorial, non-migratory *Rhinella marina* can navigate to home areas following displacements exceeding regular, natural movements, suggesting a previously unconsidered prevalence of navigational abilities amongst amphibians.

## INTRODUCTION

Many animals navigate for foraging, migration or reproduction, from tiny ants to huge whales (Allen, 2013; Wittlinger et al., 2006). Long distance navigation has been extensively studied in various species, such as sea turtles and migratory birds, offering dramatic examples of large-scale movement (Lohmann et al., 2004; Mouritsen, 2018; Mouritsen and Ritz, 2005). Such studies have been instrumental in uncovering mechanisms, like magnetoreception, that allow for accurate navigation. However, navigational abilities of many species that are largely sedentary or that live within relatively small areas are comparatively understudied. Even if these animals do not often exhibit notable navigational behaviors, they may still have homing abilities similar to those studied in organisms known for such abilities. Here, we tested the ability of the cane toad (*Rhinella marina*), a non-territorial and non-migratory nocturnal amphibian (Zug and Zug, 1979), to return to its home site following translocations exceeding typical movements.

Most studies of amphibian navigation have been conducted in migratory species (Phillips et al., 1995; Sinsch, 2006). For example, the European common toad (*Bufo bufo*) migrates up to 3 km for breeding and can return to breeding sites in straight trajectories following displacement (Sinsch, 1987). Multiple salamander species can home to breeding sites (Sinsch, 1991) and displacement studies of the Californian red-bellied newt *Taricha rivularis* show they can return to natal streams from more than 4 km (Twitty et al., 1964). More recently, Neotropical poison frogs (*Aromobatidae* and *Dendrobatidae*) have been increasingly studied for navigational abilities because of their complex parental care behaviors (Liu et al., 2019; Pašukonis et al., 2014a, 2018; Roland and O'Connell, 2015; Stynoski, 2009). Three-striped poison frog (*Ameerega trivittata*) males can navigate back to home territories following displacements of up to 800 m, exhibiting an ability to return from distances exceeding regular movements (Pašukonis et al., 2018). However, limited effort has been directed at studying navigation in anurans (frogs and toads) that do not exhibit complex spatial behaviors or the need for well-developed navigational abilities. With this study, we began to characterize space use and navigational ability in a non-territorial, non-migratory amphibian species that does not provide parental care and does not have other known life history traits suggestive of highly developed navigation.

Adult cane toads are large, nocturnal, water-breeding amphibians ubiquitous throughout their native range from the southern tip of Texas into South America (https://amphibiaweb.org; Zug and Zug, 1979; DeVore et al., 2021; Freeland and Kerin, 1991). Females lay thousands of eggs at once in a variety of water sources available in local habitats, from moving creeks and rivers to temporary shallow pools (Evans et al., 1996; Hamilton et al., 2005). Though mostly native to tropical rainforests, cane toads have gained international notoriety as an invasive species, most famously in Australia. Cane toads were introduced into northeast Australia in the 1930s and have continuously spread to encompass over 1 million square kilometers with ongoing invasive fronts expanding at up to 50 km per year (Urban et al., 2008). The variation in behaviors between native and invasive cane toads has created a dichotomy in the characterization of their spatial behaviors. Native toads and those introduced to areas not environmentally conducive for range expansion are viewed as relatively sedentary and do not perform tasks requiring precise navigation (Pettit et al., 2016; Ward-Fear et al., 2016; Zug and Zug, 1979). Toads on the invasive front in Australia are considered nomadic or dispersive (Brown et al., 2014; Schwarzkopf and Alford, 2002), and while they may move large distances, they do not seem to navigate towards a specific goal during dispersal.

Numerous studies have examined how and when invasive Australian cane toads move and the physiological characteristics affecting their space use (Brown et al., 2006; Kelehear and Shine, 2020; Phillips and Shine, 2005; Phillips et al., 2007). Though the spatial ecology of invading toads differs from that of native range toads, general activity patterns are consistent between populations (DeVore et al., 2021). However, despite a few reports on homing in cane toads (Brattstrom, 1962; Boland, 2004), no work has systematically quantified the navigational ability of cane toads using tracking methods. To characterize short-term space use and determine whether cane toads are capable of long-range navigation to a specific goal, we tracked toads and carried out translocation–homing experiments in their native rainforest habitat.

## MATERIALS AND METHODS

### Animals

The experiment was carried out around the Saut Pararé camp (4°02′N, 52°41′W) of the Nouragues Ecological Research Station in the Nature Reserve Les Nouragues, French Guiana, from January to March 2020 (the experiment was terminated early by evacuation due to the COVID-19 pandemic). The area is largely composed of primary lowland rainforest and is bordered by the Arataï river to the south.

**Fig. 1.**
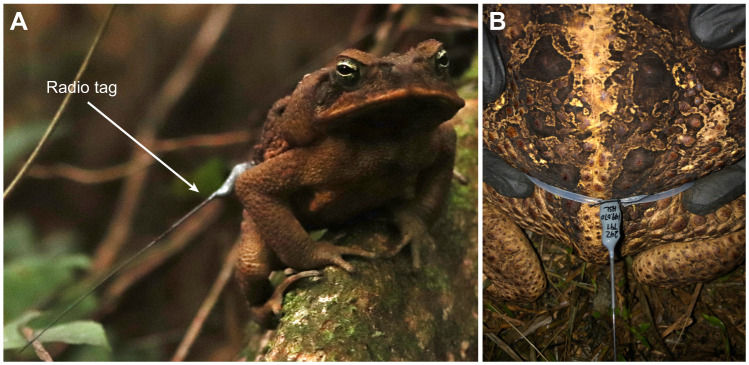
***Rhinella marina* toads tagged with radio transmitters.** (A) A male toad with an attached radio transmitter. (B) Close up of a radio transmitter attached to a toad’s waist with a belt of silicone tubing.

Toads, *Rhinella marina* (Linnaeus 1758), were mostly found by visual search at night but were also located during the day in some instances. Upon capture, toads were photographed, measured (snout to vent length, SVL), and weighed with a hanging scale (Basetech HS-51, Basetech, Winnipeg, MB, Canada). Animals were captured and tagged (Fig. 1) over a transect 300 m long near the Arataï river encompassing ∼9300 m^2^. Individuals were identified by dorsal coloration and wart patterns. A total of five females and nine males were tagged over the course of the experiment. Sex was determined by size, male release calls and recorded instances of amplexus. Five captured females weighed an average of 1.22 kg (0.57–1.71 kg) and had an average SVL of 21.8 cm (18.0–24.0 cm); nine captured males had an average mass of 0.36 kg (0.27–0.47 kg) and an average SVL of 15.0 cm (11.5–18.0 cm). Females were larger than males when measured for both SVL (*t*-test, *P*<0.05) and mass (independent 2-group Mann–Whitney *U*-test, *P*<0.05).

### Tagging and baseline tracking

A radio tag (BD2, Holohil Systems Ltd, Carp, ON, Canada) was secured to a piece of 2–4 mm silicone tubing (Dow Corning, Midland, MI, USA) and sized to sit loosely above the toad's waist and legs but tightly enough that it would not slip off (Fig. 1). The ends of the tubing were attached to form a loop around the toad using cotton thread, so that if the toad was to escape with the tag, the thread would disintegrate over time and free the toad. Total tag mass was less than 2 g and negligible compared with toad mass. Tagged animals were released at their capture site after an average of 36.9 min (18–107 min). A GPS point was recorded on a handheld GPS device (RINO655t, Garmin Ltd, Schaffhausen, Switzerland) using the waypoint averaging feature. Two toads lost tags on a total of three occasions. When they were recaptured, they were identified by dorsal wart and coloration patterns and retagged.

After release, most toads were relocated twice every 24 h during both day and night with a flexible 3-element Yagi antenna (Biotrack Ltd, Wareham, Dorset, UK) and a SIKA radio receiver (Biotrack Ltd). Effort was made to stay 2 m away from the animals, which was sometimes difficult given the brushy environment and the need to visually validate that the transmitter was attached to the animal. Some individuals were only generally localized using the antenna without visual confirmation or a specific position being recorded on some days. To account for GPS inaccuracy, toad location was recorded as a new point if the GPS plotted the spot as being at least 5 m away from the previous location of the animal. Every week, animals were captured and inspected for any tagging-related injuries. In two instances a tag was removed because of a skin injury during baseline tracking, including one female who was untagged for ∼3 weeks after an initial 3 weeks of tagging to recover from abrasions from the waistband, and was then retagged and tracked again.

**Fig. 2.**
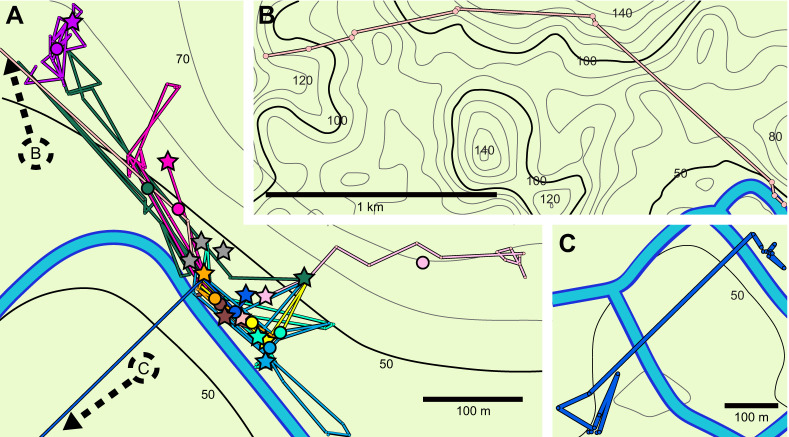
**Baseline tracking of *R. marina*.** (A) Baseline tracking for 10 toads (over 7–55 days) showing site fidelity; colors represent individuals. Location points are connected in temporal order. Larger circles represent the mean center of baseline position. Stars represent tagging locations and gray stars represent toads that were tagged but not substantially tracked. Dashed arrows point to areas represented by B and C. Contours represent 10 m changes in elevation (elevation data from Open Street Maps) and reference contour labels indicate elevation as meters above sea level. (B) One female toad did not show site fidelity and moved 2 km without translocation. (C) A male toad was initially localized near other males but was moved across the river while in amplexus.

The location of each individual was recorded over at least 7 days (both before and after translocations) (Table S1) and toads tracked for shorter durations were not included in our analyses. For our study, we considered toads to show short-term site fidelity if they showed non-unidirectional movements, repeatedly used sites and/or remained within a small, restricted area in comparison to their movement capacity. Site fidelity was determined through field observations, inspection of the individual trajectories, and the distribution of relative turn angles for each toad during baseline tracking. Clustering of relative turns around 0 deg indicates unidirectional movement, while turns around 180 deg indicates complete reversals in direction and repeated use of previously visited areas, which we also confirmed in the field. We excluded one toad (Fig. 2B; Figs S1C and S2C) out of 11 tracked for at least 7 days (seven males and four females) from translocation experiments because of unidirectional movement. Additionally, we excluded a second toad that was translocated across a river from his original home area while in amplexus (Fig. 2C; Figs S1I and S2I).

### Translocations

After animals had been tracked for at least 3 days, they were captured and translocated in the evening or night-time. After capture, toads were disoriented by walking them around in a net cage for 30–60 min prior to release at the translocation site. Six individuals (five males and one female) were translocated 500 m and five individuals (four males and one female) were translocated 1000 m. Overall, seven individuals were used in translocations (four toads experienced trials of both distances). Translocations were repeated for 500 and 1000 m within the same animal, in random sequence, although evacuation during the COVID-19 pandemic prohibited repeated sampling for all individuals. Translocation sites were varied as much as possible despite several constraints, including the river to the south, a steep hill to the north, and the need for trail access for safe nocturnal working conditions. The release site was determined from the ‘outermost’ baseline point for the individual as estimated on a local GIS map with ArcPad 10.2 (ESRI, Redlands, CA, USA) and confirmed with a GPS device in the field.

After translocation, toads were again located once during the day and once during the night in a 24 h period (Table S2). If possible, depending on the toad's proximity to the camp, multiple points were taken during the night if the animal was moving, starting in the early evening. Locations were recorded in the same manner as had been done to determine home area. Animals were given 10 days to return home. If they did not return after 10 days, they were returned home manually. Similarly, if animals exhibited any extended periods of immobility (>72 h), they were captured, and their waists checked for rubbing or wounds. One toad was injured and so the tag was removed, and the animal was released at the home site. Animals were considered to be in their home area once they were within 100 m of the translocation reference point based upon the movement range observed during baseline tracking. Following translocations, all animals were successfully untagged and released in their home areas.

### Data analysis

GPS data were uploaded into ArcGIS Pro 2 (ESRI) to visualize baseline points and homing trajectories. Temporal and spatial attributes of baseline activity and homing were calculated in R version 4.0.2 (R Foundation for Statistical Computing, Vienna, Austria) using the package ‘adehabitatLT’ (https://CRAN.R-project.org/package=adehabitatLT). Baseline range (maximum distance between two baseline points) was calculated by visually selecting and exporting outermost baseline points in ArcGIS and calculating distance in R. Total movement (cumulative path observed) was calculated by creating trajectory objects for individuals in adehabitatLT. The package ‘adehabitatHR’ (https://CRAN.R-project.org/package=adehabitatHR) was used to calculate minimum convex polygons (MCPs) for baseline data. Coordinates of the mean center of baseline activity were calculated in ArcGIS. To account for the variability in baseline tracking duration, we calculated the movement per day of observation as well as the proportion of days with larger (>10 m) movements. For these calculations, any period greater than 48 h in which toad location was not recorded was discarded. Straightness of homing trajectories was calculated by dividing the distance between the translocation release site and the point at which the toad was considered home by the cumulative movement measured. A straightness index of 1 indicates a toad that traveled in a completely straight line, with values approaching 0 indicating less direct routes taken. Normality of data was determined by Shapiro–Wilks test. Comparisons for normal data were performed with a *t*-test and comparisons of non-parametric data were performed with a Mann–Whitney *U*-test. All statistical tests of significance were performed in R (version 4.0.2).

### Permits and ethical statement

The experiments were conducted in strict accordance with French and USA laws and following the ‘Guidelines for use of live amphibians and reptiles in the field and laboratory research’ by the Herpetological Animal Care and Use Committee (HACC) of the American Society of Ichthyologists and Herpetologists (Beaupre et al., 2004) and the Association for the Study of Animal Behaviour (ASAB) ‘Guidelines for the treatment of animals in behavioral research and teaching’ (Vitale et al., 2018). This experiment was performed under approval from the scientific committee of the Nouragues Ecological Research Station. All procedures were approved by the Institutional Animal Care and Use Committee of Stanford University (protocol #33714).

## RESULTS AND DISCUSSION

### Baseline tracking

Ten toads out of 11 exhibited site fidelity during 7–55 tracking days (mean±s.d. 24±15 days). Adjusting for periods of at least 48 h in which observations of position were not made, space use was quantified over 7–32 days (18±10 days). The observed range of the 10 toads (Fig. 2A; Fig. S1) varied from 26.8 to 236.7 m (109.6±73.9 m). The sample size was not large enough to detect a sex difference. On average, toads moved more than 10 m on 60% of observation days, with many periods lacking substantial movement.

During baseline tracking, males were observed more often and in higher density than females. A minimum convex polygon encapsulating the mean centers of the seven males exhibiting site fidelity measured 858.6 m^2^. In contrast, a minimum convex polygon for the mean centers of the three females exhibiting site fidelity measured 8328.8 m^2^. Many other males were observed within the vicinity of the tracked males.

One female toad not exhibiting site fidelity (Fig. 2B; Figs S1C and S2C) was tracked for 16 days and moved a cumulative distance of 1999.4 m for a 1779.2 m displacement. Another male toad exhibited site fidelity until it was displaced more than 300 m across the river to an island by an untagged female while in amplexus (Fig. 2C; Figs S1I and S2I). The male was first observed in amplexus in its home area and relocated on the other side of the river 4 days later. The toad was untagged after 15 days on the island (of which it was in amplexus for 6 days) and did not return to its previous location for the duration of the observation.

### Translocation and homing

**Fig. 3.**
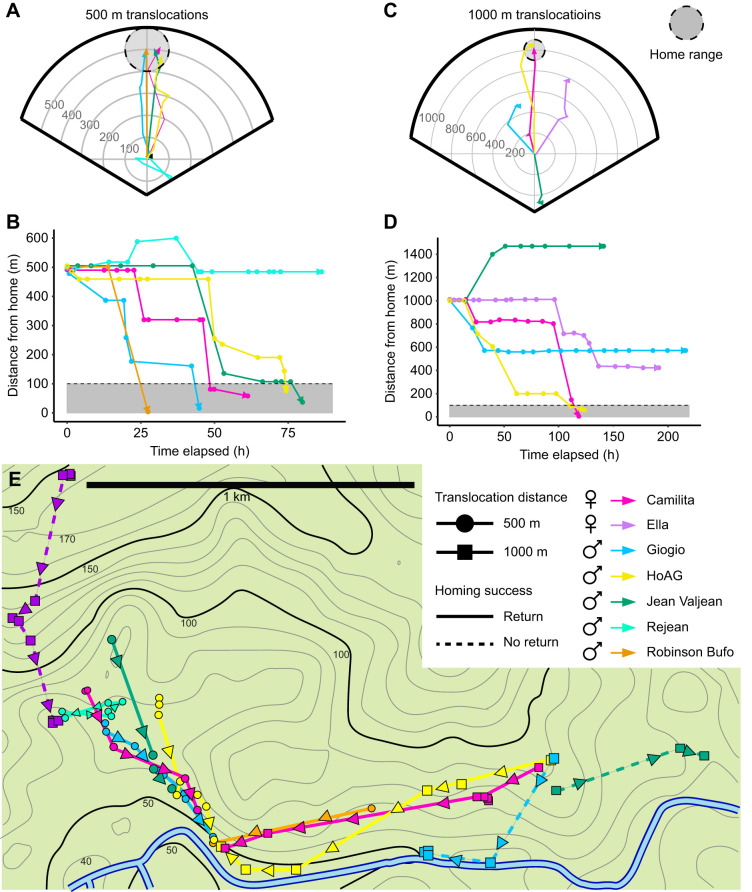
**Homing trajectories of translocated *R. marina*.** (A) Homing trajectories of toads translocated 500 m. The plot center represents translocation sites and the gray circle represents the home area. (B) Distance from home plotted against time after translocation, during homing from 500 m. The gray rectangle represents the home area. (C) Homing trajectories and (D) distance from home over time for toads translocated 1000 m. (E) Movement of toads after translocation on a map of the field site; solid lines show successful returns and dashed lines represent failure to home. Contours represent 10 m changes in elevation (elevation data from Open Street Maps) and contour labels indicate elevation as meters above sea level.

Of the six individuals translocated 500 m, five returned to their home areas within 3 days (Fig. 3A). Returns were observed between 27.5 and 79.9 h after release at the translocation site (mean±s.d. 57.5±21.6 h) (Fig. 3B). Observed straightness of four homing paths with en route relocations varied from 0.89 to 0.98 (0.94±0.04), indicating that the toads returned directly with minimal exploration. One individual did not return home, traveling 250 m in the wrong direction before returning to its translocation site. This toad was manually returned home because of observed abrasions from the belt following 2 days of no movement.

Of the five individuals translocated 1000 m, two returned to home sites within ∼5 days (Fig. 3C). Returns took 123.7 and 118.7 h (Fig. 3D). Both returned directly with measured straightness of 0.94. Both animals were observed to make their first substantial movements after 24 h post-translocation (24.25 and 26.67 h) and exhibited secondary periods of immobility following this initial movement. Two of the toads that did not return home initially moved in the correct direction before stopping prior to reaching their home areas. The remaining individual moved ∼500 m in the wrong direction before becoming stationary.

### Conclusions

The study of navigation has focused on animals that move large distances or perform tasks requiring fine scale navigation, leaving the abilities of other species capable of precise navigation overlooked. We showed for the first time that cane toads directly navigate back to home sites following translocations of up to 1 km.

Using radio-tracking of toad movements prior to translocation, we found that toads were largely sedentary without translocation, and movements were largely nocturnal, similar to previous observations in native and invasive cane toads (Carpenter and Gillingham, 1987; DeVore et al., 2021; Ward-Fear et al., 2016; Zug and Zug, 1979). This is in line with another recent study tracking cane toads in French Guiana that showed small displacements in rainforest toads when compared with coast-dwelling cane toads (DeVore et al., 2021). We did not observe larger scale foraging movements (>100 m) that have been reported in coastal toads, although the movement of a female toad for almost 2 km greatly exceeds any movements reported in coastal or rainforest sites in French Guiana. Overall, our observations generally align with previous studies, although more research is needed to gain a better understanding of cane toad movement ecology and how it covaries with biotic and abiotic factors.

During our tracking, males were clustered in relatively high density by the river, with females spread out in the surrounding area. These observations suggest female cane toads may move to areas populated by males to mate and then return to their original home areas, but detailed long-term tracking studies are needed to understand sex-specific spatial distribution and movements. Studies of invasive toads in Hawai'i have shown some sex differences in space use, but not necessarily with respect to range and total inhabited area (Ward-Fear et al., 2016). Previous tracking of native-range toads showed that the only variable affected by sex was the probability of emerging to forage, with males more likely to emerge (DeVore et al., 2021), although we did not quantify this behavior in this study. Our observations of large scale movements are comparable to those recorded in invasive toads (Schwarzkopf and Alford, 2002; Ward-Fear et al., 2016), but similar observations have not been previously reported in native-range cane toads. In Australia, the available range for expansion may contribute to their propensity to disperse (Brown et al., 2014; Phillips et al., 2006). A longer-term space use study is necessary to better characterize the full range and complexity of cane toad movement in their native habitat.

Toads showed the ability to directly navigate back to home sites following translocations of 500 and 1000 m. A previous homing study in Panama showed that cane toads could return to specific lights to hunt for insects following short distance translocations of <100 m (Brattstrom, 1962). In Australia, some cane toads could return from over a kilometer to bird ground nests they had exploited for food (Boland, 2004). Despite a very different life history and nocturnal activity, cane toads show similar navigational accuracy to poison frogs homing in the same habitat from lesser distance (Pašukonis et al., 2014b) and straighter trajectories than poison frogs moving comparable distances (Pašukonis et al., 2018), although this may be partially attributable to the lower resolution of toad trajectories. This straightness of the homing behavior contrasts with the general meandering of regular movements made by native range toads, shown in DeVore et al. (2021) and the current study (Fig. S2). Similar to poison frogs, toads were initially stationary at their translocation sites, suggesting a period of gathering bearings (Pašukonis et al., 2014a,b, 2018). Even toads that did not home successfully showed the ability to orient, with two of the three toads that did not return from 1000 m moving in the correct direction before stopping short of their observed home area. Our study period was unusually dry for the field site, and it did not rain for the duration of these translocations. Cane toads have different spatial behaviors based upon rainfall and the availability of water resources (DeVore et al., 2021), and the failure to home in our study may potentially have been due to stopping at available water resources. Overall, toads demonstrated a capacity to return with accuracy to their home areas from distances that exceeded their observed regular movements and ranges.

The sensory mechanisms that allow for this homing are unknown. Magnetoreception and olfaction have been shown to play an important role in navigation in other bufonid species in smaller scale translocations (Sinsch, 1990, 1987, 1992). It is possible that auditory taxis to male calling contributed to homing as males were largely congregated in one area. However, males were not calling throughout the entire study, and it is unlikely that calls would travel a kilometer through dense forest. The use of visual cues is unlikely given that toads did not explore the area and the dense rainforest understory results in many obstacles. Simple olfactory taxis to water also seems unlikely in the case of successfully returning animals, given that toads could have returned to stretches of river closer to their translocation sites (Fig. 3E). More likely is that multiple sensory modalities are involved and contribute to various stages of navigation. The potential for the use of multiple navigation mechanisms (beaconing, path integration, etc.) has been described in amphibians (Sinsch, 2006), and more research is needed to identify which of these paradigms are applicable to the cane toad's apparent ability to navigate. In addition, translocations exceeding the distance of maximum observed movements (in this case ∼2 km) should be executed to determine whether toads rely on previous experiences to navigate.

Our observations show that cane toads are capable of navigation over long distances after displacement from a home area, suggesting navigational abilities may be widely shared among amphibians. Cane toads are common, large and invasive amphibians able to carry biologging devices (such as accelerometers and GPS), making them particularly interesting for field and lab studies on amphibian navigation. Future research could include testing toad navigation during manipulation of sensory systems to identify which sensory cues are important for navigation, as well as identifying the neural basis of navigation in amphibians.
